# Multi-Trait Single-Step GBLUP Improves Accuracy of Genomic Prediction for Carcass Traits Using Yearling Weight and Ultrasound Traits in Hanwoo

**DOI:** 10.3389/fgene.2021.692356

**Published:** 2021-07-30

**Authors:** Hossein Mehrban, Masoumeh Naserkheil, Deukhwan Lee, Noelia Ibáñez-Escriche

**Affiliations:** ^1^Department of Animal Science, Shahrekord University, Shahrekord, Iran; ^2^Department of Animal Science, University College of Agriculture and Natural Resources, University of Tehran, Karaj, Iran; ^3^Department of Animal Life and Environment Sciences, Hankyong National University, Gyeonggi-do, South Korea; ^4^Institute for Animal Science and Technology, Universitat Politècnica de València, València, Spain

**Keywords:** single-step GBLUP, genomic prediction, accuracy, ultrasound measurement, genetic correlation, Hanwoo

## Abstract

There has been a growing interest in the genetic improvement of carcass traits as an important and primary breeding goal in the beef cattle industry over the last few decades. The use of correlated traits and molecular information can aid in obtaining more accurate estimates of breeding values. This study aimed to assess the improvement in the accuracy of genetic predictions for carcass traits by using ultrasound measurements and yearling weight along with genomic information in Hanwoo beef cattle by comparing four evaluation models using the estimators of the recently developed linear regression method. We compared the performance of single-trait pedigree best linear unbiased prediction [ST-BLUP and single-step genomic (ST-ssGBLUP)], as well as multi-trait (MT-BLUP and MT-ssGBLUP) models for the studied traits at birth and yearling date of steers. The data comprised of 15,796 phenotypic records for yearling weight and ultrasound traits as well as 5,622 records for carcass traits (backfat thickness, carcass weight, eye muscle area, and marbling score), resulting in 43,949 single-nucleotide polymorphisms from 4,284 steers and 2,332 bulls. Our results indicated that averaged across all traits, the accuracy of ssGBLUP models (0.52) was higher than that of pedigree-based BLUP (0.34), regardless of the use of single- or multi-trait models. On average, the accuracy of prediction can be further improved by implementing yearling weight and ultrasound data in the MT-ssGBLUP model (0.56) for the corresponding carcass traits compared to the ST-ssGBLUP model (0.49). Moreover, this study has shown the impact of genomic information and correlated traits on predictions at the yearling date (0.61) using MT-ssGBLUP models, which was advantageous compared to predictions at birth date (0.51) in terms of accuracy. Thus, using genomic information and high genetically correlated traits in the multi-trait model is a promising approach for practical genomic selection in Hanwoo cattle, especially for traits that are difficult to measure.

## Introduction

There is a growing interest in improving economically important traits in beef cattle breeding programs worldwide because it has a substantial effect on the overall profitability of the meat production system. Carcass traits are a comprehensive term that constitutes a major set of target traits for genetic evaluation and selection in beef cattle. In the Korean beef industry, Hanwoo cattle has been intensively bred for meat over the last four decades, and its beef is well known for its marbled fat, tenderness, juiciness, flavor (Jo et al., 2012), and is regarded as a healthy food option (Joo et al., 2017). Presently, the selection index in Hanwoo breeding programs mainly focuses on carcass traits, including backfat thickness (BFT), carcass weight (CW), eye muscle area (EMA), and marbling score (MS), which are major selection criteria in attempts to improve both the quantity and quality of meat (Kim et al., 2017). However, measurement of these traits for genetic evaluation is expensive and difficult, as it requires the animals to be slaughtered to record their phenotypes. An efficient way to evaluate carcass traits is the use of ultrasound measurements at yearling age, which has the potential to improve the rate of genetic progress and reduce the time and expenses of slaughter tests (Jansen et al., 1985; Wilson, 1992). Previous studies have reported high positive genetic correlations between ultrasound measurements in yearling bulls and heifers and the corresponding traits measured in the carcass of progeny (Moser et al., 1998; Reverter et al., 2000; Crews and Kemp, 2002; Kemp et al., 2002; Crews et al., 2003; Lee and Kim, 2004; MacNeil et al., 2010; Yokoo et al., 2015; Elzo et al., 2017). In other words, the evaluation of animals based on ultrasounds of yearlings would be an alternative to anticipate the decision-making about selection for carcass traits. Thus, the inclusion of ultrasound data as correlated traits along with genomic information can result in improved accuracy in the estimation of breeding values for carcass traits.

In recent decades, with the advent of high-throughput single nucleotide polymorphism (SNP) genotyping technologies, genomic selection has become an appealing tool for accelerating genetic improvement in many breeding programs throughout the world (Meuwissen et al., 2001). Genomic selection allows for a better prediction of EBVs than the classical methods, especially for young animals without performance. Several genomic methods have been developed and applied, among which single-step genomic BLUP (ssGBLUP) has been widely used in routine genomic evaluation (Legarra et al., 2009; Misztal et al., 2009; Aguilar et al., 2010; Christensen and Lund, 2010). A single-step genomic BLUP is a modification of the GBLUP method, in which phenotypes of genotyped and non-genotyped individuals are used simultaneously along with an **H** matrix that combines the pedigree-based numerator relationship matrix (**A**) and a genomic relationship matrix (**G**) (Legarra et al., 2009; Aguilar et al., 2010; Christensen and Lund, 2010). Several studies have indicated that this single-step method leads to higher accuracy than the pedigree-based BLUP or genomic BLUP (GBLUP) in Hanwoo cattle (Lee et al., 2017; Mehrban et al., 2019; Lopez et al., 2020; Naserkheil et al., 2020) and other beef cattle (Onogi et al., 2014; Lourenco et al., 2015; Elzo et al., 2017). Most of these studies have been based on single-trait analyses, although this method is suitable for multi-trait analyses. Previously, it has been shown that a multi-trait genomic model for genetically correlated traits can increase the accuracy of breeding values compared to single-trait genomic models using simulated (Calus and Veerkamp, 2011; Guo et al., 2014) and real data (Jia and Jannink, 2012; Ismael et al., 2017; Mehrban et al., 2019; Song et al., 2019). In our previous study (Mehrban et al., 2019), the strong positive genetic correlation between yearling weight and CW resulted in an improvement in the accuracy of genomic prediction for CW in Hanwoo when using multi-trait genetic analyses. Furthermore, based on the literature, it is expected that the use of yearling weight and ultrasound data can improve the accuracy of genomic prediction for carcass traits while simultaneously reducing the generation interval (Elzo et al., 2017; Mehrban et al., 2019). Nonetheless, utilizing all available yearling ultrasound measurements to further increase the accuracy of genomic prediction for corresponding carcass traits in Hanwoo cattle has not yet been investigated. Hence, the objective of this study was to assess the improvement in the accuracy of genetic predictions for carcass traits by using yearling measurements on weight and ultrasound traits as well as genomic information in Hanwoo beef cattle. To this aim four models: pedigree-based single-trait BLUP (ST-BLUP), pedigree-based multi-trait BLUP (MT-BLUP), single-trait single-step genomic BLUP (ST-ssGBLUP), and multi-trait single-step genomic BLUP (MT-ssGBLUP) were compared, based on bias, dispersion, and accuracy of breeding values for carcass traits using a linear regression (LR) method (Legarra and Reverter, 2018).

## Materials and Methods

### Animals and Phenotypes

The phenotypic measurements used in this study were derived from 10,114 bulls and 5,682 Hanwoo steers born between 1997 and 2017 at Hanwoo Experiment Station (Seosan, South Korea).

The records of body weight at 12 months of age for 15,796 animals, yearling ultrasound traits of 8,945 animals, carcass traits of 5,622 steers, and a pedigree consisting of 54,284 animals were used.

The eight traits analyzed were yearling body weight (YW), ultrasound backfat thickness (UBFT), ultrasound eye muscle area (UEMA), ultrasound intramuscular fat (UIMF), BFT, CW, EMA, and MS. Briefly, carcass traits were measured according to the Korean carcass grading system in steers at approximately 24 months of age, ribbed between the 13^th^ rib and the first lumbar vertebrae after chilling for approximately 24 h postmortem. The MS was graded using a 9-point scale following the Korean Beef Marbling Standard (1 = trace, 9 = very abundant). The ultrasound carcass traits were collected by an experienced technician using a B-mode real-time ultrasound device (HS-2000, FHK Co. Ltd, Japan) with an 18 cm, 3.5 MHz linear probe. The animals were scanned for the longissimus muscle area between the 13^th^ rib and the first lumbar UBFT over the longissimus muscle at point three-fourths the length ventrally of the longissimus muscle area, and UIMF was the percentage intramuscular fat. Phenotypic data for UBFT, UEMA, and UIMF were obtained by analyzing the ultrasonic images with scanning software developed by HIC based on the CUP Lab at Iowa State University^1^. The yearling weight for each animal was determined from the weight at the termination of the test (body weight at ∼12 months) and the previous weight recorded at a time point before the termination (body weight at ∼6 months), according to the equation described by Park et al. (2013). Descriptive statistics are shown for each trait in Table 1.

**TABLE 1:** Descriptive statistics for the ultrasound at 12 months of age, carcass traits, and yearling weight in Hanwoo cattle.

**Trait (unit)**	**n**	**Mean (SE)**	**min**	**max**	**SD**	**CV (%)**
UIMF (%)	8943	2.57 (0.02)	0.10	13.40	1.43	55.57
UEMA (cm^2^)	8945	54.11 (0.08)	24.20	88.70	7.90	14.59
UBFT (mm)	8945	3.68 (0.01)	1.10	9.10	1.05	28.55
BFT (mm)	5622	9.92 (0.05)	1.00	35.00	3.95	39.83
CW (kg)	5619	370.48 (0.57)	213.00	562.00	42.80	11.55
EMA(cm^2^)	5617	81.62 (0.12)	50.00	121.00	8.98	11.00
MS (score)	5622	3.53 (0.02)	1.00	9.00	1.64	46.50
YW (kg)	15796	357.13 (0.35)	190.49	547.65	44.07	12.34

*UIMF, ultrasound of intramuscular fat; UEMA, ultrasound of EMA; UBFT, ultrasound BFT; BFT, backfat thickness; CW, carcass weight; EMA, eye muscle area; MS, marbling score; YW, yearling weight. SE: standard error; SD: standard deviation; CV: coefficient of variation.*

### Genotypes

A total of 6,616 animals were genotyped (animal and SNP call rate > 90%) with the Illumina Bovine SNP50 BeadChip (Illumina, San Diego, CA, United States). These genotyping data were from animals (4,284 steers and 2,332 bulls) with at least one observation for the interest of traits or as a sire in the pedigree. Missing genotypes of SNPs were imputed using FImpute V3 software (Sargolzaei et al., 2014), and 52,791 SNPs on the 29 chromosomes were obtained. Markers with minor allele frequencies lower than 0.01 (8,819 SNPs), and a maximum difference between the observed and expected frequency of 0.15, as a departure of heterozygous from the Hardy-Weinberg equilibrium (23 SNPs) were excluded. After quality control, the number of SNPs remaining for subsequent analyses was 43,949.

### Statistical Analyses

#### Estimation of Variance-Covariance Components

The Bayesian multi-trait pedigree-based animal model was applied to obtain variance-covariance components for YW, ultrasound, and carcass traits using the gibbsf90test software (Misztal et al., 2015) as follows:

(1) y=Xb+Zu+e

where **y** is the vector of observations for the trait of interest; **b** is the vector of fixed effects, including batch-sex-technician (108 levels), birth place (103 levels), and age of recording as a covariate for ultrasound traits; batch-sex (87 levels) and birth place (109 levels) for YW, slaughter date (274 levels), and slaughter age (days from birth to slaughter) was considered as a covariate for carcass traits; **u** is the vector of random genetic additive effects; **e** is the vector of random residual effects; **X** and **Z** are incidence matrices related to fixed and random genetic additive effects, respectively. Var(**u**) = **G**⊗**A** and Var(**e**) = **R**⊗**I** were assumed, where **A** is the numerator relationship matrix, **I** is the identity matrix, and **G** and **R** are additive genetic and residual covariance, respectively, for the eight traits.

The Markov Chain Monte Carlo (MCMC) method was used to estimate variance components and heritabilities in 550,000 cycles with a thinning interval of 50 and 50,000 iterations as burn-in. The convergence of the chain was verified by visual inspection of trace plots. The variance components and correlations were estimated as the posterior means of the corresponding sampled values. Furthermore, the estimated variance components from the multi-trait model were compared with those obtained using a single-trait animal model, which assumes zero for genetic and residual covariance among the traits.

### Modeling Methods

#### Pedigree-Based BLUP

Pedigree-based evaluations were performed to estimate breeding values for each trait based on ST-BLUP and MT-BLUP models. The model is defined in equation (1). Furthermore, the genetic and residual covariances for the eight traits were assumed to be zero for the ST-BLUP model.

#### Single-Step GBLUP

The ssGBLUP analyses using both ST-ssGBLUP and MT-ssGBLUP models were applied for genetic evaluations, which combined pedigree and genomic information. The ssGBLUP has the same model as BLUP, except for the inverse of numerator relationship matrix **A** ^– 1^, which was replaced by matrix **H** ^– 1^. This matrix was obtained using the following equation (Aguilar et al., 2010, 2011) with preGSf90 software (Aguilar et al., 2014):

(2) $${\text{H}}^{-1}={\text{A}}^{-1}+\left[\begin{array}{cc} 0 & 0\\ 0 & {\left(0.95\,\text{G}+0.05\,{\text{A}}_{22}\right)}^{-1}-{\text{A}}_{22}^{-1} \end{array}\right]$$

where **A** is the numerator relationship matrix, **G** is the genomic relationship matrix (VanRaden, 2008), and **A**_22_ is the numerator relationship matrix for genotyped animals.

Variance components used in the MT-BLUP (ST-BLUP) and MT-ssGBLUP (ST-ssGBLUP) were estimated using the pedigree-based multi-trait (single-trait) animal model (Equation 1). Breeding values in all models were obtained using BLUPF90 software (Misztal et al., 2015).

#### Genetic Evaluation at Birth and Yearling Date

Genetic evaluation of animals can be performed at two stages of birth or yearling dates (Kim et al., 2017), regardless of whether single- or multi-trait models are considered and with or without genomic information. Note that the phenotype data of steers obtained at slaughter date (around two years of age), but the body weight and ultrasound measurements were also recorded at the yearling date for each animal. In traditional BLUP evaluations, the EBV of carcass traits for a steer can be expressed as the parent average EBV at birth date and the composition of parent average and the yield deviation (due to correlated traits) at the yearling date (Mrode, 2014). In addition, genomic information contains direct genomic value and pedigree prediction derived from **A**_22_ (Aguilar et al., 2010). Hence, the extra source of information to estimate breeding values at the yearling date relative to the birth date is the part of the yield deviation, regardless of the model. By comparing the two evaluations at birth and yearling dates, increases in accuracy due to including correlated traits (yearling measurement of weight and ultrasound) of focal animals in the models can be observed.

#### Assessment of Accuracy, Bias, and Dispersion

There are several validation methods to evaluate genetic prediction (Legarra and Reverter, 2018); however, a validation method that can support any model and any data structure is preferable. Recently, Legarra and Reverter (2018) described the ability of the LR method to validate the results obtained from genetic evaluations. In this method, estimates of bias, dispersion, and accuracy of EBV/GEBV derived from a dataset containing old (“partial”) compared with a dataset containing both old and new records (“whole”) for the same individuals. The validation population was defined as animals born in 2016 and 2017; therefore, the EBV/GEBV were estimated for focal animals in either the whole and partial dataset using different models. In the partial dataset, the phenotypes of focal animals were assumed to be unknown, and only the genotypes and pedigree information were retained. The number of steers in the focal population was 803 for carcass traits, 815 for ultrasound traits, and 817 for YW. The expectation of EBV/GEBV accuracy from the partial dataset is $\rho_{w,p}=\sqrt{\frac{\text{cov}\,\left({\hat{u}}_{\text{w}},{\hat{u}}_{\text{p}}\right)}{\left(1+\bar{\text{F}}+2\,\bar{\text{f}}\right)\,\sigma_{\text{u},\infty }^{2}}}$, where $\bar{\text{F}}$ is the average inbreeding coefficient, 2$\bar{\text{f}}$ is the average relationship between individuals, $\sigma_{\text{u},\infty }^{2}$ is the genetic variance at equilibrium in a population under selection, which is estimated using Gibbs sampling technique described by Sorensen et al. (2001), and ${\hat{\text{u}}}_{\text{w}}$(${\hat{\text{u}}}_{\text{p}}$) is the vector of estimated breeding value for focal animals using the whole (partial) dataset (Legarra and Reverter, 2018). Estimator $\frac{1}{\rho_{p_{i},p_{M\,T-s\,s\,G\,B\,L\,U\,P}}}$ represents the relative increase in accuracy from the i^th^ method to MT-ssGBLUP in the partial data (Legarra and Reverter, 2018). Thus, $\frac{1}{\rho_{p_{i},p_{M\,T-s\,s\,G\,B\,L\,U\,P}}}-1$ is the superiority of accuracy using MT-ssGBLUP compared to the i^th^ method in all traits under study when partial data were used. This value confirms the extent to which the inclusion of genomic information and correlated traits in the MT-ssGBLUP method increases the accuracy of predictions for steers.

The expected bias is estimated from the difference between the mean estimated breeding value in partial and whole data, $\mu_{w,p}=\bar{{\hat{\text{u}}}_{\text{p}}}-\bar{{\hat{\text{u}}}_{\text{w}}}$. The expected value of this estimator is zero if the evaluation is unbiased. The estimator of dispersion of the breeding value is the regression of ${\hat{\text{u}}}_{\text{w}}$ on ${\hat{\text{u}}}_{\text{p}}$, $b_{w,p}=\frac{\text{cov}\,\left({\hat{u}}_{\text{w}},{\hat{u}}_{\text{p}}\right)}{v\,a\,r\,\left({\hat{u}}_{\text{p}}\right)}$. In the absence of over- or under-dispersion of breeding values, the expected value of this estimator is one (Legarra and Reverter, 2018).

## Results

### Estimates of Genetic Parameters

The variance components and heritability estimates were similar for both single and multi-traits except for CW, in which the magnitude of genetic variance in the multi-trait model was significantly higher than in the single-trait, which led to increasing heritability in the former (0.42) relative to the latter (0.31) model. The estimated heritabilities were moderate for the ultrasound and YW ranged from 0.19 to 0.33, while the heritabilities were high for BFT, EMA, and MS traits ranged from 0.48 to 0.59 (Supplementary Table 1). The lowest and highest heritability values were obtained for UIMF and MS, respectively, regardless of whether a single- or multi-trait model was used.

Favorable and high genetic correlations were estimated between UIMF and MS (0.78), UBFT and BFT (0.63), and UEMA and EMA (0.65). In addition, the estimated genetic correlation between YW and CW was positive and high (0.84) and relatively moderate between YW and EMA (0.43) (Supplementary Table 2). The phenotypic correlations were generally lower than the genetic correlations between UIMF and MS (0.42), UBFT and BFT (0.40), UEMA and EMA (0.48), and YW and CW (0.78) (Supplementary Table 2).

### Genetic Evaluation of Steers

**FIGURE 1:**
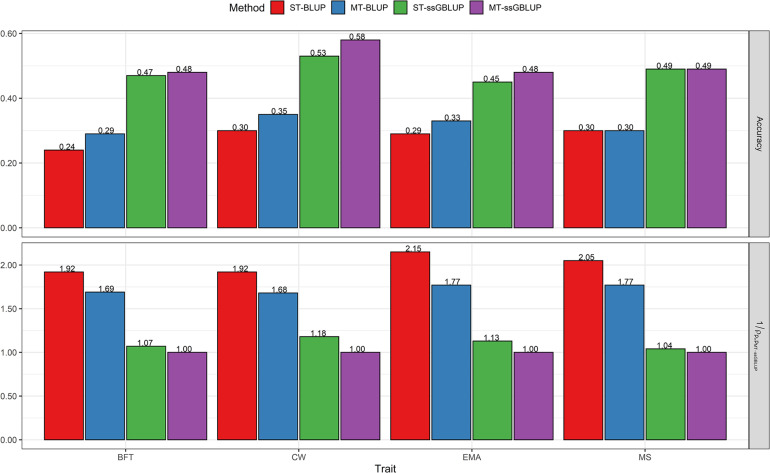
Accuracy of breeding values obtained using BLUP (ST-BLUP, MT-BLUP) and ssGBLUP (ST-ssGBLUP, MT-ssGBLUP) models. $\frac{1}{\rho_{p_{i},p_{M\,T-s\,s\,G\,B\,L\,U\,P}}}$: the relative increase of accuracy from i^th^ model to MT-ssGBLUP in the partial data for steers at birth date. ST and MT are single-trait and multi-trait analyses, respectively. BFT, backfat thickness; CW, carcass weight; EMA, eye muscle area; MS, marbling score; YW, yearling weight.

**FIGURE 2:**
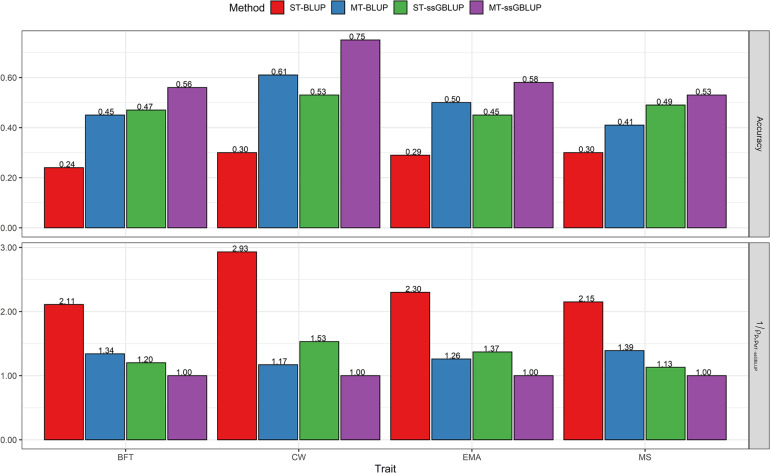
Accuracy of breeding values obtained using BLUP (ST-BLUP, MT-BLUP) and ssGBLUP (ST-ssGBLUP, MT-ssGBLUP) models. $\frac{1}{\rho_{p_{i},p_{M\,T-s\,s\,G\,B\,L\,U\,P}}}$: the relative increase of accuracy from i^th^ model to MT-ssGBLUP in the partial data for steers at yearling date. ST and MT are single-trait and multi-trait analyses, respectively. BFT, backfat thickness; CW, carcass weight; EMA, eye muscle area; MS, marbling score; YW, yearling weight.

Predictive accuracies for carcass traits at birth and yearling dates of steers obtained with the four models (ST-BLUP, MT-BLUP, ST-ssGBLUP, and MT-SSBLUP) are presented in Figures 1 and 2. Our results indicate that averaged across all traits, the accuracy of ssGBLUP methods (0.52) was higher than that of pedigree-based BLUP (0.34), regardless of the use of single- or multi-trait models. On average, the accuracy of prediction can be further improved by implementing yearling weight and ultrasound data in the MT-ssGBLUP model (0.56) for the corresponding carcass traits compared to the ST-ssGBLUP model (0.49).

### Genetic Evaluation of Steers at Birth Date

**FIGURE 3:**
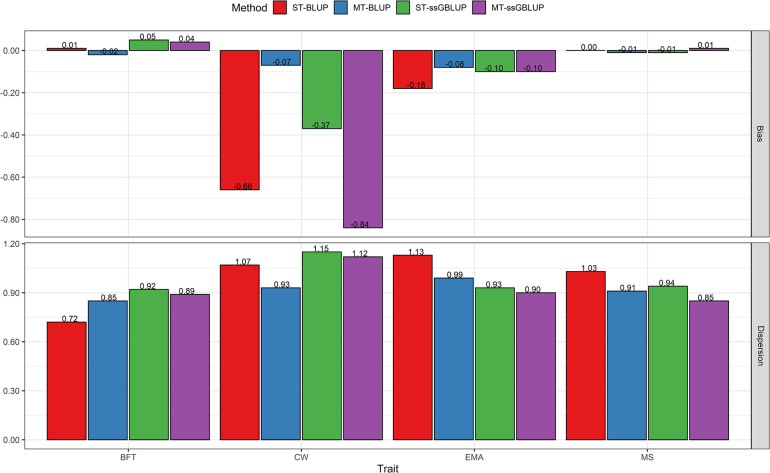
Bias and Dispersion of breeding values obtained using BLUP (ST-BLUP, MT-BLUP) and ssGBLUP (ST-ssGBLUP, MT-ssGBLUP) models for steers at birth date. ST and MT are single-trait and multi-trait analyses, respectively. BFT, backfat thickness; CW, carcass weight; EMA, eye muscle area; MS, marbling score; YW, yearling weight.

The results showed that the accuracies of estimated breeding values from single-step genomic BLUP methods were substantially higher than those of conventional BLUP for all traits, regardless of the single or multi-trait models. When information on the correlated traits was included in the prediction of breeding values in the multi-trait analyses, the accuracy increased for BFT, CW, and EMA, whereas a trivial gain in accuracy was observed for MS by changing from a single-trait to a multi-trait model (Figure 1). Our results show that the MT-ssGBLUP approximately doubled (100 % improvement) the accuracy of breeding values compared with the ST-BLUP model, and the superiority over MT-BLUP was obvious for BFT (69%), CW (68%), EMA (77%), and MS (77%) in the present study. Moreover, the accuracy was improved by 7% for BFT, 18% for CW, 13% for EMA, and 4% for MS by using the MT-ssGBLUP method rather than the ST-ssGBLUP (Figure 1). Concerning the bias, for genetic predictions of validation animals, pedigree BLUP methods showed less bias than predictions from ssGBLUP methods for BFT, while bias for MS was small and similar in pedigree and genomic methods. The smallest and largest biases for CW were observed with the MT-BLUP and MT-ssGBLUP methods, respectively. The prediction obtained with ST-BLUP for EMA was marginally more biased compared with the other three models (Figure 3). For all traits, the average value for the estimator of dispersion varied from 0.72 to 1.13 (average absolute deviation 0.99) for ST-BLUP, from 0.85 to 0.99 (average absolute deviation 0.92) for MT-BLUP, from 0.92 to 1.15 (average absolute deviation 0.98) for ST-ssGBLUP, and from 0.85 to 1.12 (average absolute deviation 0.94) for the MT-ssGBLUP method (Figure 3).

### Genetic Evaluation of Steers at Yearling Date

**FIGURE 4:**
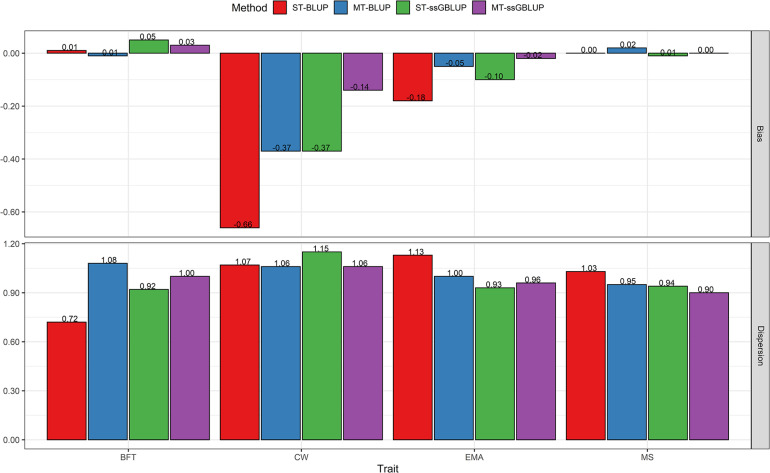
Bias and Dispersion of breeding values obtained using BLUP (ST-BLUP, MT-BLUP) and ssGBLUP (ST-ssGBLUP, MT-ssGBLUP) models for steers at yearling date. ST and MT are single-trait and multi-trait analyses, respectively. BFT, backfat thickness; CW, carcass weight; EMA, eye muscle area; MS, marbling score; YW, yearling weight.

Figure 2 compares the results obtained with ST-BLUP, MT-BLUP, ST-ssGBLUP, and MT-ssGBLUP for carcass traits when predictions were based on data of the correlated traits at the yearling age of steers. Accuracies ranged from 0.24 to 0.30 (average 0.28) for ST-BLUP, from 0.41 to 0.61 (average 0.49) for MT-BLUP, from 0.45 to 0.53 (average 0.49) for ST-ssGBLUP, and from 0.53 to 0.75 (average 0.61) for the MT-ssGBLUP method. Across the studied traits, the multi-trait models substantially outperformed the single-trait models in both pedigree and genomic evaluations. The highest accuracies were obtained when using MT-ssGBLUP for all traits at the yearling date. The results show that the relative gain in accuracy of GEBV using MT-ssGBLUP was twice as high as that estimated with ST-BLUP for BFT, EMA, and MS, whereas for CW it was approximately triple. In addition, the MT-ssGBLUP method was superior to MT-BLUP with an increase or relative gain of 34% for BFT, 17% for CW, 26% for EMA, and 39% for MS. In addition, using the MT-ssGBLUP model led to an increase in accuracy of 20, 53, 37, and 13% for BFT, CW, EMA, and MS, respectively, compared with the ST-ssBLUP model (Figure 2). In terms of the GEBV bias of the validation individuals at the yearling date, the MT-ssGBLUP had the lowest bias for CW and EMA, while the pedigree-based BLUP methods showed the lowest bias for the BFT trait (Figure 4). The bias estimates for MS were not very different among the models except for MT-BLUP, in which the highest bias was observed (Figure 4). Across the studied traits, the value of dispersion ranged from 0.72 to 1.13 (average absolute deviation 0.99) for ST-BLUP, from 0.95 to 1.08 (average absolute deviation 1.02) for MT-BLUP, from 0.92 to 1.15 (average absolute deviation 0.98) for ST-ssGBLUP, and from 0.90 to 1.06 (average absolute deviation 0.98) for MT-ssGBLUP method (Figure 4).

### Comparisons of Accuracy, Bias, and Dispersion Between Evaluation at Birth and Yearling Date of Steers

The results of evaluations at birth date were comparable with those obtained from the yearling date in terms of accuracy, bias, and dispersion of predictions. When additional information of the correlated traits at the yearling date was used, prediction accuracies were higher than those of birth date for all traits (Figures 1, 2). The results showed that the accuracies of EBV/GEBV at the yearling date were higher than those derived from multi-trait models at birth. The population accuracy of GEBVs at yearling date compared with birth date were 0.56 vs. 0.48 for BFT, 0.75 vs. 0.58 for CW, 0.58 vs. 0.48 for EMA, and 0.53 vs. 0.49 for MS using the MT-ssGBLUP method. In other words, this study showed the impact of genomic information and the correlated traits on predictions at yearling date (average 0.61) using the MT-ssGBLUP model, which was advantageous compared to predictions at birth date (average 0.51) in terms of accuracy. In addition, the results of the MT-BLUP model at yearling (birth) date were 0.45 (0.29), 0.61 (0.35), 0.50 (0.33), and 0.41 (0.30) for BFT, CW, EMA, and MS, respectively (Figures 1, 2). Regarding the bias, predictions based on genomic information and the correlated traits at the yearling date using multi-trait models appeared to have less bias than predictions at birth date, except for CW in the MT-BLUP model (Figures 3, 4). The results of dispersion using multi-trait models showed values close to 1 at the yearling date for all traits when compared to birth date (Figures 3, 4).

## Discussion

Based on the observed results, the yearling ultrasound and carcass traits were moderate to high heritable traits, which were comparable to those reported in Brangus (Moser et al., 1998), Angus (Reverter et al., 2000), Simmental (Crews et al., 2003), Nellore (Yokoo et al., 2015; Silva et al., 2021), multi-breed Angus-Brahman (Elzo et al., 2017), and Hanwoo cattle (Lee and Kim, 2004; Hwang et al., 2014; Choi et al., 2015). Moreover, the heritability estimates for carcass traits and yearling weight in our study corresponded to those obtained by Mehrban et al. (2019) in Hanwoo cattle. Furthermore, our results showed moderate to strong positive genetic correlations between yearling ultrasound and corresponding carcass traits, which were within the range of values estimated in the literature (Moser et al., 1998; Reverter et al., 2000; Kemp et al., 2002; Crews et al., 2003; Lee and Kim, 2004; Elzo et al., 2017; Su et al., 2017). These results indicate that better responses to selection for carcass traits can be expected when the corresponding carcass traits are measured by ultrasound at yearling age.

The results of this study showed that the accuracies of breeding values obtained from ssGBLUP were substantially higher than those of the traditional BLUP model for all traits at birth and yearling date, regardless of using single- or multi-trait genomic models. The observed gain in accuracy is due to additional variation in genomic information capture in Mendelian sampling by a realized relationship matrix (Christensen et al., 2012). Hence, it was noted that the gain in accuracy from pedigree to genomic predictions can be explained by better relationships (VanRaden, 2008; Hayes et al., 2009). Consequently, the inclusion of genomic information in the single-trait analyses led to an average increase of accuracy from 0.28 to 0.49 by changing the method from ST-BLUP to ST-ssGBLUP across all carcass traits at birth or yearling date. Similarly, results of the multi-trait methods showed an increase in accuracy of 0.32 to 0.51 at birth date and from 0.49 to 0.61 at yearling date by moving the method from MT-BLUP to MT-ssGBLUP for all the carcass traits. Many studies have reported the superiority of ssGBLUP over pedigree-based methods in both single (Tsuruta et al., 2011; Carillier et al., 2014; Onogi et al., 2014; Koivula et al., 2015; Lourenco et al., 2015; Lee et al., 2017; Teissier et al., 2018; Oget et al., 2019; Lopez et al., 2020; Mehrban et al., 2021) and multi-trait models (Mehrban et al., 2019; Karaman et al., 2020; Lopez et al., 2020).

The accuracies of ST-BLUP models for carcass traits were similar to those previously reported for Hanwoo beef cattle (Lee et al., 2017; Mehrban et al., 2019; Lopez et al., 2020; Mehrban et al., 2021); however, the accuracies derived from ST-ssGBLUP models were lower than those obtained by Lopez et al. (2020) and higher than those obtained by Lee et al. (2017) and Mehrban et al. (2019). One of the main reasons for this difference between the studies could be the difference in the proportion of genotyped individuals as a reference population (Goddard and Hayes, 2009; VanRaden et al., 2009). The number of genotyped steers for carcass traits in previous studies in the same breed was 988 (Lee et al., 2017), 1,151 (Mehrban et al., 2019), and 16,892 (Lopez et al., 2020), while in this study it was 4,284. Moreover, the accuracies of the ST-ssGBLUP model for carcass traits were higher than those obtained by Mehrban et al. (2021), except for CW using the same dataset. This difference between the results of the two studies is due to the cross-validation method. Mehrban et al. (2021) used fold-cross validation with precorrected data that could be biased (Legarra and Reverter, 2018). The LR method was addressed in this study, without pre-corrected data. This method is independent of heritability and errors in estimates of fixed effects (Legarra and Reverter, 2018), in addition to the application of any statistical model and any data structure (Misztal et al., 2020). For instance, Bermann et al. (2021) applied the LR method to analyze mortality in broiler chickens using a threshold model and showed that this method is a useful tool for predicting improvement in the accuracy of breeding values due to the inclusion of genomic information.

One effective strategy to improve the accuracy of evaluations for carcass traits that are difficult or expensive to measure is the use of a multi-trait model with indicator traits that are easier or cheaper to measure. One of the main benefits of the multi-trait model is the simultaneous use of information from relatives and genetically correlated traits, mostly for the candidate animals and their offspring without any phenotypes (e.g., the pre-selection of young bulls for progeny testing).

The results demonstrated that multi-trait models generally yielded higher accuracies than single-trait models in both pedigree and genomic evaluations. Based on our results, the average improvement of the accuracies for carcass traits was +4 percentage points (from 0.28 to 0.32%) using MT-BLUP instead of ST-BLUP, and +2 percentage points (from 0.49 to 0.51) by changing the model from ST-ssGBLUP to MT-ssGBLUP when using the information of correlated traits from relatives at the birth date of steers. Similar to predictions at the birth date, accuracies using multi-trait models were higher than single-trait models for all traits at the yearling date because of the additional information of correlated traits (yearling weight and ultrasound traits) recorded from steers. In other words, when the data for yearling ultrasound of steers as correlated traits were available at yearling age, the average gain in accuracy for carcass traits obtained +21 percentage points (from 0.28 to 0.49) by changing the model from ST-BLUP to MT-BLUP, and +12 percentage points (from 0.49 to 0.61) by moving the model from ST-ssGBLUP to MT-ssGBLUP. Consistent with our results, several previous studies have shown that multi-trait genomic prediction is superior to single-trait genomic analyses using simulated (Calus and Veerkamp, 2011; Guo et al., 2014) and real data (Jia and Jannink, 2012; Schulthess et al., 2016; Ismael et al., 2017; Mehrban et al., 2019; Song et al., 2019; Karaman et al., 2020).

In general, the advantage of using multi-trait models for improving the prediction accuracies at the yearling date was more obvious than those obtained at the birth date for all the considered traits. These results can be attributed to the use of phenotypic records from YW and ultrasound traits of steers (in the validation population) in addition to their relatives’ information at the yearling date, whereas information from the correlated traits (YW and ultrasound traits) is not available for focal animals at birth, and only information from their relatives was included in the multi-trait models. In this line, (Ismael et al., 2017) revealed that using a multi-trait model improved the reliability of breeding values for an interval from calving to first high activity, while no gain was observed for calving to first insemination, resulting in the number of phenotypic records being higher for those calving to first insemination (1,472,313) than those calving to first high activity (36,504). Similarly, Song et al. (2019) reported that the ssGBLUP model performed better than both the pedigree-based BLUP or GBLUP for seven body measurement traits in pigs. They demonstrated an improvement in accuracy from a two-trait ssGBLUP model compared to ST-ssGBLUP when a high genetic correlation between traits was observed. Moreover, a similar conclusion was drawn by Sun et al. (2010), who reported that the reliability of EBV for fertility traits (low heritability) was increased with the multi-trait traditional BLUP model when information regarding milk yield traits (high heritability) as a genetically correlated trait was used in Danish Holstein cattle. Similar to the findings of Mehrban et al. (2019), the prediction accuracy was improved for CW and EMA by switching from a single-trait to multi-trait model; however, for two other traits (BFT and MS), the results of that study disagree with our findings using the multi-trait model. This difference can be explained by the existence of a high genetic correlation between BFT and UBFT (0.63), and MS and UIMF (0.78), along with the inclusion of ultrasound measurements on the candidate animals in the multi-trait model. Lopez et al. (2020) indicated that accuracies from single-trait and multi-trait models were similar for carcass traits, which is inconsistent with the results of the present study. However, they did not consider YW and ultrasound traits in the model and found low to moderate genetic correlations among carcass traits. Consequently, the results of the current study demonstrated that the genetic gain in carcass traits can be improved at birth and yearling date when yearling weight and ultrasound traits are included as correlated traits in a multi-trait model.

## Conclusion

The findings revealed that using genomic information along with including high genetically correlated traits, such as yearling weight and ultrasound traits, in the multi-trait model can be useful in ongoing Hanwoo cattle breeding programs, especially for traits that are difficult to measure, such as carcass traits.

## Data Availability Statement

The data analyzed in this study is subject to the following licenses/restrictions: This dataset was generated to select proven bulls by Hanwoo Improvement center under the national government rule of Hanwoo breeding schemes. Requests to access these datasets should be directed to DL.

## Ethics Statement

All animals used in this study were cared for slaughtered according to animal health and welfare guidelines approved by the Animal Care and Use Committee of the National Institute of Animal Science (NIAS), Rural Development Administration of South Korea. Genotype and phenotype data were collected by Hanwoo Improvement Center (HIC) of National Agricultural Cooperative Federation.

## Author Contributions

HM and DL conceived and designed the study. HM conducted statistical analyses. HM and MN interpreted and discussed the results and drafted the manuscript. DL was responsible for phenotypic and genotyping data collection. NI-E conceived the study, evaluated the experiments, interpreted, and discussed the results, and edited the manuscript. All authors read and approved the final manuscript.

## Conflict of Interest

The authors declare that the research was conducted in the absence of any commercial or financial relationships that could be construed as a potential conflict of interest.

## Publisher’s Note

All claims expressed in this article are solely those of the authors and do not necessarily represent those of their affiliated organizations, or those of the publisher, the editors and the reviewers. Any product that may be evaluated in this article, or claim that may be made by its manufacturer, is not guaranteed or endorsed by the publisher.

## Footnotes

1
https://www.cuplab.com

## Abbreviations

HIC: Hanwoo Improvement Center
ST-BLUP: Single-trait pedigree best linear unbiased prediction
MT-BLUP: Multi-trait pedigree best linear unbiased prediction
GBLUP: Genomic best linear unbiased prediction
ssGBLUP: Single-step genomic best linear unbiased prediction
SNP: Single nucleotide polymorphism
GEBV: Genomic estimated breeding value
BFT: Backfat thickness
CW: Carcass weight
EMA: Eye muscle area
MS: Marbling score
YW: Yearling body weight
UBFT: Ultrasound backfat thickness
UEMA: Ultrasound eye muscle area
UIMF: Ultrasound intramuscular fat
LR: Linear regression.
