# Supplementary material for: Multi-Trait Single-Step GBLUP Improves Accuracy of Genomic Prediction for Carcass Traits Using Yearling Weight and Ultrasound Traits in Hanwoo
Source: Front Genet. 2021 Jul 30;12:692356. doi: 10.3389/fgene.2021.692356 (PMC8363309; doi:10.3389/fgene.2021.692356)
Supplement: Supplementary file 1 [file Data_Sheet_1.docx]

Supplementary Material

**Supplementary Table 1.** Variance components and heritability (95% HPD) estimated from pedigree and phenotypic information in the Hanwoo breeding population

|  | Model | $\boldsymbol{\sigma}_{\boldsymbol{a}}^{\boldsymbol{2}}$ | $\boldsymbol{\sigma}_{\boldsymbol{e}}^{\boldsymbol{2}}$ | $\boldsymbol{\sigma}_{\boldsymbol{p}}^{\boldsymbol{2}}$ | $\boldsymbol{h}^{\boldsymbol{2}}$ |
| --- | --- | --- | --- | --- | --- |
| UIMF | ST | 0.24  (0.17-0.31) | 0.96  (0.91-1.02) | 1.20  (1.16-1.24) | 0.20  (0.15-0.25) |
|  | MT | 0.23  (0.18-0.28) | 0.98  (0.93-1.03) | 1.21  (1.17-1.25) | 0.19  (0.15-0.23) |
| UEMA | ST | 10.61  (8.56-12.64) | 21.35  (19.77-23.02) | 31.96  (30.88-33.06) | 0.33  (0.28-0.39) |
|  | MT | 9.98  (8.14-11.94) | 20.72  (19.19-22.18) | 30.70  (29.71-31.72) | 0.32  (0.27-0.38) |
| UBFT | ST | 0.21  (0.17-0.26) | 0.48  (0.45-0.52) | 0.69  (0.67-0.72) | 0.30  (0.25-0.36) |
|  | MT | 0.21  (0.17-0.25) | 0.48  (0.45-0.51) | 0.69  (0.67-0.71) | 0.30  (0.25-0.36) |
| BFT | ST | 7.26  (5.98-8.60) | 5.51  (4.49-6.52) | 12.77  (12.20-13.31) | 0.57  (0.48-0.66) |
|  | MT | 7.15  (5.84-8.45) | 5.59  (4.54-6.57) | 12.74  (12.18-13.30) | 0.56  (0.47-0.65) |
| CW | ST | 339.28  (251.10-438.00) | 761.41  (681.30-841.70) | 1100.69  (1055.00-1146.40) | 0.31  (0.23-0.39) |
|  | MT | 596.32  (478.30-714.50) | 828.31  (732.60-921.90) | 1424.63  (1358.50-1482.90) | 0.42  (0.35-0.49) |
| EMA | ST | 30.45  (24.02-36.87) | 32.92  (27.83-37.95) | 63.37  (60.67-66.22) | 0.48  (0.40-0.57) |
|  | MT | 34.60  (27.82-41.80) | 34.40  (28.77-39.76) | 68.99  (65.70-72.01) | 0.50  (0.41-0.59) |
| MS | ST | 1.44  (1.21-1.70) | 1.03  (0.83-1.21) | 2.47  (2.36-2.58) | 0.58  (0.50-0.67) |
|  | MT | 1.43  (1.17-1.69) | 0.97  (0.78-1.18) | 2.40  (2.29-2.51) | 0.59  (0.51-0.69) |
| YW | ST | 366.61  (300.30-432.30) | 781.81  (730.90-831.10) | 1148.42  (1117.50-1180.50) | 0.32  (0.27-0.37) |
|  | MT | 354.09 (293.60-422.30) | 792.65 (742.90-840.80) | 1146.74  (1114.60-1177.10) | 0.31  (0.26-0.36) |

UIMF, ultrasound of intramuscular fat; UEMA, ultrasound of EMA; UBFT, ultrasound BFT; BFT, backfat thickness; CW, carcass weight; EMA, eye muscle area; MS, marbling score; YW, yearling weight. ST, MT, $\sigma_{a}^{2}$, $\sigma_{e}^{2}, \sigma_{p}^{2}, h^{2}$, and HPD: single-trait analysis, multi-trait analysis, additive genetic variance, error variance, phenotypic variance, heritability, and the highest posterior density, respectively. Numbers in parentheses are the lower and upper 95% highest posterior densities, respectively.

**Supplementary Table 2.** Estimates of genetic (above diagonals), and phenotypic (below diagonals) correlations among ultrasound and carcass traits in Hanwoo cattle

|  | UIMF | UEMA | UBFT | BFT | CW | EMA | MS | YW |
| --- | --- | --- | --- | --- | --- | --- | --- | --- |
| UIMF | 1 | 0.04 (0.07) | 0.46 (0.06) | 0.11 (0.08) | 0.14 (0.07) | 0.28 (0.07) | 0.78 (0.04) | -0.05 (0.07) |
| UEMA | 0.05 (0.01) | 1 | 0.16 (0.07) | -0.09 (0.07) | 0.40 (0.06) | 0.65 (0.05) | -0.07 (0.08) | 0.41 (0.05) |
| UBFT | 0.15 (0.01) | 0.22 (0.01) | 1 | 0.63 (0.05) | -0.01 (0.07) | -0.12 (0.07) | 0.14 (0.07) | 0.15 (0.07) |
| BFT | 0.07 (0.01) | 0.08 (0.02) | 0.40 (0.01) | 1 | 0.07 (0.07) | -0.25 (0.07) | 0.03 (0.07) | -0.05 (0.07) |
| CW | 0.04 (0.01) | 0.42 (0.01) | 0.15 (0.01) | 0.27 (0.02) | 1 | 0.56 (0.05) | 0.17 (0.07) | 0.84 (0.02) |
| EMA | 0.09 (0.01) | 0.48 (0.01) | 0.05 (0.01) | 0.01 (0.02) | 0.57 (0.01) | 1 | 0.35 (0.06) | 0.43 (0.06) |
| MS | 0.42 (0.01) | 0.01 (0.02) | 0.08 (0.02) | 0.08 (0.02) | 0.12 (0.02) | 0.24 (0.02) | 1 | 0.03 (0.07) |
| YW | 0.05 (0.01) | 0.51 (0.01) | 0.23 (0.01) | 0.18 (0.02) | 0.78 (0.01) | 0.40 (0.01) | 0.06 (0.02) | 1 |

UIMF, ultrasound of intramuscular fat; UEMA, ultrasound of EMA; UBFT, ultrasound BFT; BFT, backfat thickness; CW, carcass weight; EMA, eye muscle area; MS, marbling score; YW, yearling weight. Numbers in parentheses are the standard deviations of the posterior densities.
